# Sparstolonin B suppresses free fatty acid palmitate‐induced chondrocyte inflammation and mitigates post‐traumatic arthritis in obese mice

**DOI:** 10.1111/jcmm.17099

**Published:** 2021-12-24

**Authors:** Haiwei Ma, Chenglong Xie, Gaolu He, Zhengtai Chen, Hongwei Lu, Hongqiang Wu, Hancheng Cai, Zihan Dai, Baolong Li, Cong Xu, Enxing Xue

**Affiliations:** ^1^ Department of Orthopaedic Surgery The Second Affiliated Hospital and Yuying Children’s Hospital of Wenzhou Medical University Wenzhou China; ^2^ Department of Clinical Medicine Second Clinical Medical College Wenzhou Medical University Wenzhou China

**Keywords:** free fatty acid, inflammation, NF‐κB, osteoarthritis, sparstolonin B, toll‐like receptor 4

## Abstract

Abnormal lipid metabolism, such as systemic increased free fatty acid, results in overproduction of pro‐inflammatory enzymes and cytokines, which is crucial in the development of obesity‐related osteoarthritis (OA). However, there are only a few drugs that target the lipotoxicity of OA. Recent researches have documented that the traditional Chinese medicine, Sparstolonin B (Ssn B), exerted anti‐inflammatory effects in various diseases, but not yet in OA. On the basis of this evidence, our works purposed to evaluate the effect of Ssn B on free fatty acid (FFA) palmitate (PA)‐stimulated human osteoarthritic chondrocytes and obesity‐associated mouse OA model. We found that Ssn B suppressed PA‐triggered inflammatory response and extracellular matrix catabolism in a concentration‐dependent approach. *In vivo*, Ssn B treatment inhibited cartilage degeneration and subchondral bone calcification caused by joint mechanical imbalance and alleviated metabolic inflammation in obesity. Mechanistically, co‐immunoprecipitine and molecular docking analysis showed that the formation of toll­like receptor 4 (TLR4)/myeloid differentiation protein‐2 (MD‐2) complex caused by PA was blocked by Ssn B. Subsequently, it leads to inactivation of PA‐caused myeloid differentiation factor 88 (MyD88)‐dependent nuclear factor‐kappaB (NF‐κB) cascade. Together, these findings demonstrated that Ssn B is a potential treatment agent for joint degenerative diseases in obese individuals.

## 1 INTRODUCTION

Osteoarthritis (OA), one of the commonest joint degenerative illness, is typified by cartilage loss, subchondral bone sclerosis as well as osteophyte formation.^1^ A variety of underlying factors, including ageing, obesity, trauma, being female and congenital malformation, have obviously exhibited the potential to escalate the risk of OA.^2^, ^3^, ^4^, ^5^ Among these risk contributors, obesity seems to be great significance.^6^ Elevated mechanical stress on the joint platform is recognized as one predisposing factor in obesity‐related OA; however, it is likely not the only one.^7^, ^8^ Recent investigations have demonstrated that obesity‐linked free fatty acids (FFAs), for example, palmitic acid (PA) and stearic acid (SA), were considered as novel pro‐inflammatory factors and caused chondrocyte dysfunction and even death.^9^, ^10^ Moreover, in individuals with OA, the content of FFAs is elevated in the joint fluid, synovium and articular cartilage.^11^, ^12^, ^13^

Toll‐like receptors (TLRs) have been correlated with multiple diseases; nonetheless, accumulating evidences opine that TLR4 participates in the pathogenesis of osteoarthritis.^14^, ^15^, ^16^ For obesity people, PA, as a kind of damage­associated molecular patterns (DAMPs), was considered a novel agent for TLR4 activation.^16^, ^17^ However, the process of PA and TLR4 protein binding requires myeloid differentiation‐2 (MD‐2) protein as an auxiliary, which is involved in PA‐mediated TLR4 complex formation through crosstalking with the TLR4 extracellular domain.^18^ After the formation of the PA‐TLR4‐MD‐2 complex, MyD88, IL­1­receptor­associated kinases (IRAKs) as well as TNF receptor‐associated factor 6 (TRAF6) are recruited to promote the transcription of p65 and activate nuclear factor kappa B (NF­κB) pathway.^16^ Finally, p65 in the nucleus binds to the promoter site of the target gene, triggering a cascade of catabolic and inflammatory reactions.^19^ Therefore, specifical inhibition of the TLR4/MD‐2 cascade might be identified as a therapeutic schedule with great promise for OA, especially in obese individual.

Sparstolonin B (Ssn B) is an natural compound isolated form the Chinese herb *Sparganium stoloniferum* (*S*.*stoloniferum*), whose tubers have long been applied in traditional Chinese medicine (TCM).^20^ The Ssn B was identified as a selective TLR antagonist, showing significant anti‐tumour, anti‐obesity and anti‐inflammatory effects in various diseases.^20^, ^21^, ^22^, ^23^, ^24^, ^25^, ^26^ It could inhibit lipopolysaccharide (LPS)‐mediated macrophages’ inflammation by selective blockage of TLR4/NF‐κB axis^20^ and suppresses leptin‐induced inflammation and lipid accumulation in hepatic Kupffer cells by decreasing TLR4 trafficking.^23^ Moreover, Ssn B was reported to inhibit the LPS caused production of cytokines in 3T3‐L1 adipocytes, as well as reduce high‐fat diet‐stimulated obesity in rats.^25^ In addition, Ge et al. in recent study revealed that the Ssn B exerts a protective effect in the rat intervertebral disc degenerative model by targeting TLR4/MyD88/NF‑κB‐triggered inflammation.^27^ Nonetheless, whether the Ssn B participates in the modulation of joint degenerative disease like osteoarthritis is not well elucidated. Based on the literature, the Ssn B was a potent anti‐inflammatory agent and has a closed relation to the lipid metabolism.^25^ Herein, we firstly verified that Ssn B alleviated the inflammation level and ECM degradation of human osteoarthritis chondrocytes exposed to palmitate and explained its potential molecular mechanism. Moreover, the Ssn B‐induced protection *in vivo* was evaluated by mouse obesity‐related OA model.

## 2 MATERIALS AND METHODS

### 2.1 Reagents and antibodies

Sparstolonin B (purity >98%), palmitate and type II collagenases were supplied by Sigma‐Aldrich (St Louis, USA). Abcam (Cambridge, UK) provided the primary antibodies against TLR4, MyD88, TRAF6, Lamin B1, iNOS along with GADPH. Bioworld (OH, USA) supplied the anti‐IRAK1, goat anti‐rabbit, as well as anti‐mouse IgG‐HRP, whereas Cell Signaling Technology (CST, Danvers, USA) provided the antibodies against COX‐2, p65, as well as IκBα. MD‐2 antibody was from eBioscience (CA, USA). Jackson ImmunoResearch (West Grove, PA, USA) supplied us with the Alexa Fluor^®^488‐conjugated and Alexa Fluor^®^594‐conjugated goat anti‐rabbit IgG (H+L) secondary antibody. In addition, DAPI was supplied by Beyotime (SH, China). Finally, Gibco (Grand Island, USA) supplied the cellular growth materials.

### 2.2 Isolation of human primary osteoarthritic chondrocytes

Ethics Committee of the Second Affiliated Hospital of Wenzhou Medical University approved the tissue collection procedure and was as per Declaration of Helsinki guidelines.^28^ Besides, the study subjects gave an informed consent. We acquired the knee cartilage samples from 6 participants consisting of 3 men and 3 women (aged 65–73 years) who received TKR (total knee replacement). The hyaline cartilage tissue was collected and chopped as much as possible and soaked in a DMEM/F12 solution of type II collagenase (2 mg/mL) at 37°C for 4 hours. After washing and centrifuging with PBS, added DMEM/F12 medium containing 10%FBS and 1%antibiotic to resuspend the cells and seeded them in a six‐well plate. To prevent loss of phenotype, we select chondrocytes within two generations for subsequent experiments.

### 2.3 Cell experiment strategy

To explore protective influences of diverse concentrations of Ssn B, we exposed the cells to 500 μM PA, in combination with Ssn B pretreatment at varied levels (3, 10 and 30 μM), or alone. Moreover, our control group was not treated except medium replacement. Chondrocytes were pretreated with twenty‐four hours of Ssn B. As for the evaluation of NF‐κB signal activity, the exposure time of PA was shortened to 2 h. While to examine the functional parameters, consisting of ECM marker, or inflammatory, we extended the duration to 24 h.

### 2.4 Cell viability analysis

The Dojindo Co‐CCK‐8 kit (Japan) was employed to explore the Ssn B cytotoxicity on the chondrocytes, as described in the manufacturer‐provided protocol. In brief, the 2nd passage chondrocyte was inoculated in 96‐well plates (8000/well) for 24 hours, and then incubation in different concentration of Ssn B (0, 1, 3, 10, 30 and 100 μM) performed for 24 hours or 48 hours. After the specified time is over, we exhausted the medium in each well and introduced 100μl of DMEM/F12 enriched with 10 μl of CCK‐8 reagent to all the wells of the plate and then incubated at 37°C for an additional 2 hours. Finally, Thermofisher spectrophotometer was employed to determine the absorbance at 450 nm.

### 2.5 Measurement of inflammatory factors and secreted proteins

Nitric oxide interaction in growth medium was assayed by the Griess reagent as documented previously.^29^ The level of PGE2, TNF‐α, aggrecan, IL‐6, ADAMTS‐5, collagen II, as well as MMP‐13 in the supernatants of the cell cultures was measured with the commercial ELISA kits (R&D Systems, Minneapolis, MN) as described by the manufacturer.

### 2.6 Western blot analysis

After the treatment, the intracellular proteins were extracted by RIPA lysate, and then the protein concentration was detected by the BCA assay kit (Beyotime, China) to prepare the equivalent samples (40 μg). Each sample was fractionated in SDS/PAGE gels and blotted onto the Bio‐Rad PVDF membranes (USA). Subsequently, 5% dry milk was employed to block the membranes for 2 h. Afterwards, we inoculated the membranes with the primary antibody against Lamin B (1:5000), TLR4 (1:500), iNOS (1:1000), TRAF6 (1:1000), COX‐2 (1:1000), p65 (1:1000), IκBα (1:1000), MD‐2 (1:1000), MyD88 (1:1000), IRAK1 (1:500), as well as GADPH (1:5000) and incubated overnight at 4°C. Thereafter, we washed the membrane three times with TBST and incubated in the secondary antibody for 2 hours at RT (room temperature). Thereafter, the films were rinsed thrice using TBST, and Invitrogen‐electrochemiluminescence plus reagent employed to view the blots. The Bio‐Rad Image Lab software was finally employed to determine blot's intensity.

### 2.7 Immunofluorescence

As for cellular fluorescence staining, the cells were seeded on glass slides and treated with the above‐mentioned agents. Then, the glass slides were washed thrice with PBS, then fixed with paraformaldehyde and then permeabilized with 0.1% Triton X‐100. Afterwards, 10% goat serum (dissolved in PBS) was employed to block non‐specific antigens of each sample. Thereafter, PBS was employed to rinse the glass slides, which were inoculated by primary antibodies: collagen Ⅱ (1:200), p65 (1:200), as well as MMP‐13 (1:200) at 4°C overnight. Rinsing of glass plates was done the next day and then inoculated with Alexa Fluor^®^488‐labelled secondary antibodies (diluted in PBS, 1:300) for 1h at RT, followed by labelling by using DAPI for 5 min. Random selection of five fields for every slide was conducted for observation using the Olympus fluorescence microscope (Tokyo, Japan).

### 2.8 Immunoprecipitation

We performed RIPA lysate to extract cellular protein and added enough TLR4 antibody to incubate for one hour. After incubation, added magnetic beads to enrich the immune complex in a 4°C overnight. Thereafter, ice‐cold PBS was employed to rinse the precipitates four times and then boiled the sample buffer to release the proteins. Finally, the content of MD‐2 in the sample is detected by western blot.

### 2.9 Molecular modelling

We performed ChemBioDraw to form the Ssn B’s structure. ChemBio3D was employed in minimizing its energy. We downloaded the crystal structure of the human MD‐2/lipid IVa complex (PDB code 2E59) from the Protein Data Bank. By normalized with PyMoL, the default settings were employed in determining the lowest energy conformations of docking. The AutoDock Tools provides the ligand binding flexibility with the binding pocket residues. In addition, it was employed to perform the protein‐ligand docking assessment. Finally, we employed the UCSF PyMoL and the Ligplot+software to generate the 3D and 2D view images, respectively.

### 2.10 Animal model

Our experimental animals are seven‐week‐old B6 female wild‐type mice. A total of 40 mice are used, with 10 mice in each group, all of which were purchased from the Animal Research Center of the Chinese Academy of Science (Shanghai). All of the experimental procedures were approved by the Animal Care and Use Committee of Wenzhou Medical University. Figure 5A indicated overall timeline of our animal experiments. Starting when the mice were seven‐week old, standard diet (STD) or high‐fat diet (HFD) was fed to the animals, of which 60% of the energy came from fat (Research Diets, USA). In addition, mice were weighed every two weeks. After 3 months, all mice received destabilization of the medial meniscus (DMM) surgery on their right knee joints. Meanwhile, mice in one of the groups were fed an STD or HFD and received an intraperitoneal injection of 5 mg/kg of Ssn B daily, and those in the other group were fed an STD or HFD and treated with an equal volume of saline by intraperitoneal injection. Notably, based on the previous research, we selected the concentration of 5 mg/kg/day to explore the protective effect of Ssn B *in vivo*.^23^, ^25^, ^30^, ^31^, ^32^ As for DMM surgery, in brief it entails cutting the tendon between tibial plateau and the medial meniscus to achieve meniscal instability.^33^ The mice were allowed unlimited activity along with ad libitum access to food and water for 12 weeks.

### 2.11 X‐ray assay

After 12 weeks of surgery, animals received an X‐ray. Knee joint X‐ray was produced by the digital X‐ray machine (Kubtec Model XPERT.8; KUB Technologies Inc) at 160 μA and 50 kV.

### 2.12 Safranin O staining

Knee tissue is collected and fixed with 4% paraformaldehyde for one day. Afterwards, the samples were decalcified by 10% EDTA solution for one month. After dehydration with gradient alcohol, the tissue was embedded in paraffin and then cut into 5 μm slices. For each joint sample, we selected a slice every 50 μm, a total of 10 slices in total, and performed SO staining. Finally, our histological researchers accessed the degree of cartilage degeneration, according to the Osteoarthritis Research Society International (OARSI) scoring system.^33^, ^34^ Briefly, OARSI scoring system was described as 0 = no damage; 1 = roughened articular surface and small fibrillations; 2 = fibrillation down to the layer immediately below the superficial layer and some loss of surface lamina; 3 = loss of surface lamina and fibrillations extending down to the calcified cartilage; 4 = major fibrillations and cartilage erosion down to the subchondral bone; 5 = major fibrillations and erosion of up to 80% of the cartilage and 6 = more than 80% loss of cartilage. Tibial plateau and femoral condyle of the joint were scored separately. The summed score represented the additive scores for each quadrant of the joint. This method of analysis enabled assessment of severity of lesions as well as reflecting the surface area of cartilage affected with OA lesions.

### 2.13 ELISA test of mouse serum

To explore the contents of IL‐1β, TNF‐α, as well as IL‐6 in serum of mice, the blood specimens collected. After centrifugation to remove cells and cell debris, the ELISA kits were used to detect the levels of the above‐mentioned inflammatory factors in the serum.

### 2.14 Statistical analyses

Our experiments were conducted at least 5 times. These data are indicated by mean ± SD. SPSS software program 20.0 was employed to analyse the data. Data were assessed with one‐way analysis of variance (ANOVA) and the n by Tukey's test for comparison between control and treatment groups. *p *< 0.05 were considered significant.

## 3 RESULTS

### 3.1 Influences of Ssn B on human chondrocytic viability

**FIGURE 1:**
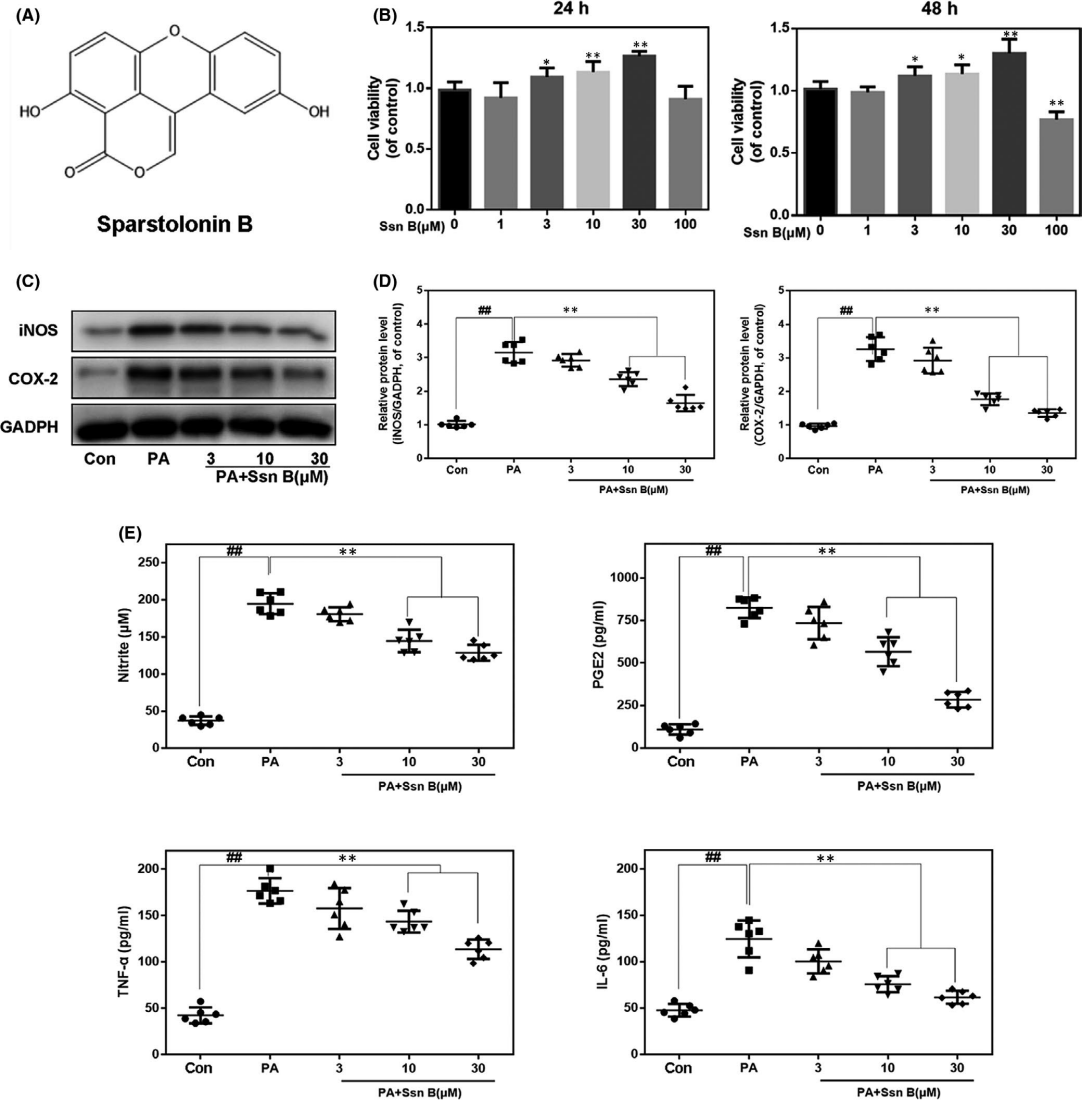
Ssn B inhibits PA‐induced inflammatory effect on chondrocyte. (A) Chemical structure of Sparstolonin B. (B) The viability of chondrocytes after Ssn B treatment for 24h or 48h, detected by CCK‐8. (C, D) The expression of iNOS and COX‐2 proteins in chondrocytes treated as above, detected by WB. (E, F) The production of nitric oxide, PGE2, TNF‐α and IL‐6 from chondrocytes treated as above, detected by ELISA and Griess reagent. The data present as averages ± SD. ##*p*<0.01, **p<0.01, *n* = 6

Chemical structure of Ssn B is shown in Figure 1A. The cytotoxic effect of Ssn B on chondrocytes was assayed at an ascending concentration of Ssn B (0, 1, 3, 10, 30 and 100 μM) for 24 and 48 hours with the CCK‐8 kit. Chondrocytic viability increased after Ssn B treatment at 3, 10 and30 μM, but decreased when the Ssn B concentration increased to 100 μM (Figure 1B). Therefore, 3, 10 or 30 µM Ssn B was employed in the following experiments.

### 3.2 Influences of Ssn B on the expressions of inflammatory factors in PA‐mediated human osteoarthritic chondrocytes

In order to explore whether Ssn B represses the inflammation in PA‐treated human osteoarthritic chondrocytes, diverse‐related biomarkers were assayed. Western blot was used to assess the production of iNOS and COX‐2. In Figure 1C and D, Ssn B inhibited the PA‐induced upregulation of iNOS and COX‐2 proteins with a concentration‐dependent approach. Besides, PA stimulation caused the endogenous production of PGE2 and nitric oxide. But, Ssn B addition reduced PGE2 and nitric oxide overproduction in a concentration‐dependent approach (Figure 1E). Additionally, after Ssn B treatment, a dose‐dependent suppression of TNF‐α and IL‐6 generation was reported in the ELISA analyses (Figure 1E). These findings demonstrated the anti‐inflammatory effect of Ssn B (all *p *< 0.05).

### 3.3 Influences of Ssn B on ECM degradation in PA‐stimulated human osteoarthritic chondrocytes

**FIGURE 2:**
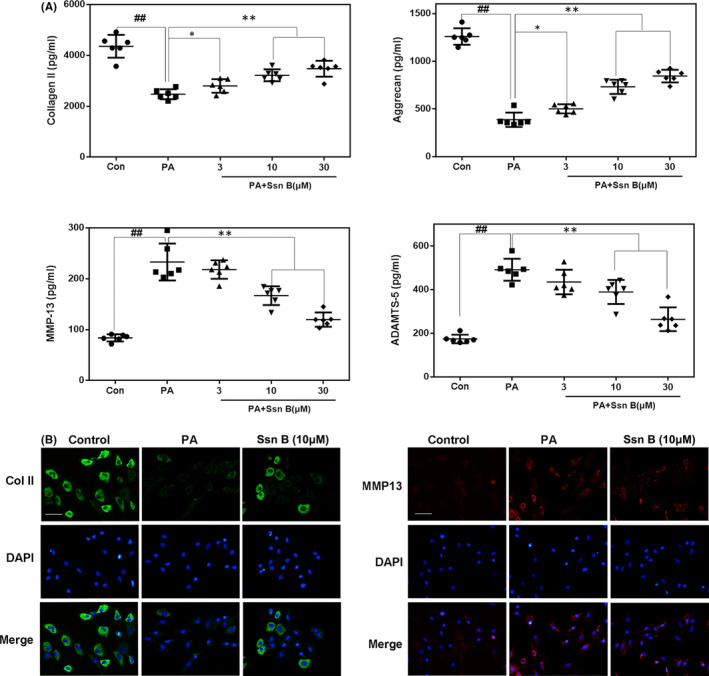
Ssn B inhibits PA‐mediated ECM degradation in chondrocytes. (A) The production of collagen II, aggrecan, MMP‐13 and ADAMTS‐5 proteins from chondrocytes treated as above, detected by ELISA. (B) The fluorescence of collagen II and MMP‐13 in chondrocytes treated as above (scale bar: 50 μm). The data present as averages ± SD. ##*p*<0.01, *p < 0.05, **p < 0.01, *n* = 6

Then, we evaluated the role of Ssn B on PA‐caused chondrocytic ECM degradation. In Figure 2A, Ssn B elevated the expression of collagen II and aggrecan, but repressed MMP‐13 and ADAMTS‐5 production in a concentration‐dependent manner, compared with PA‐stimulated group. Besides, collagen II and MMP‐13 immunofluorescence results of chondrocytes were consistent with the ELISA results (Figure 2B). Altogether, these results reveal that Ssn B alleviates ECM degradation in PA‐stimulated chondrocytes.

### 3.4 Influences of Ssn B on PA‐mediated NF‐κB activation in PA‐stimulated human osteoarthritic chondrocytes

**FIGURE 3:**
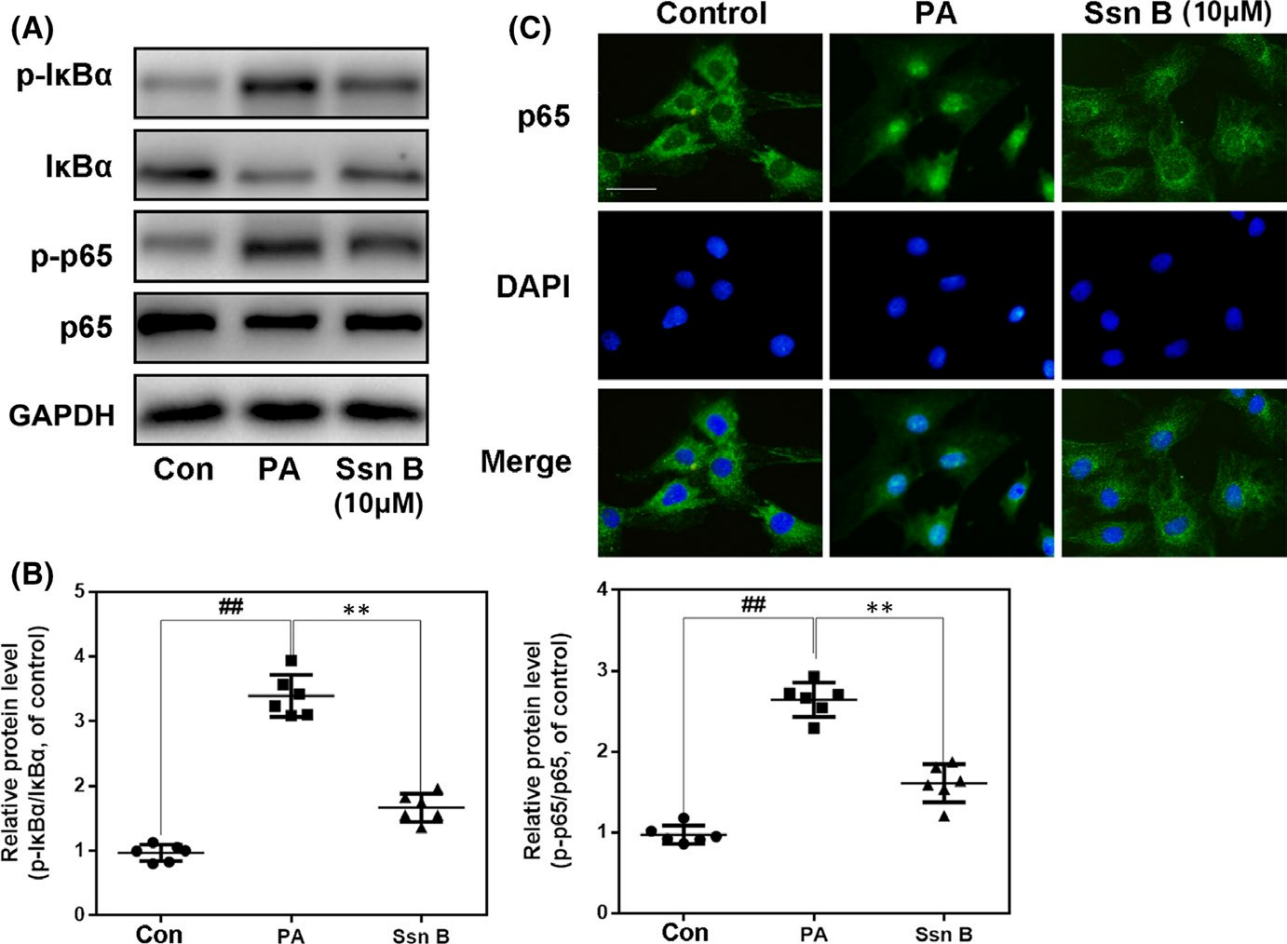
Effect of Ssn B on PA‐mediated NF‐κB signal activation. (A, B) The expression of p‐IκBα, IκBα, p‐p65 and p65 proteins in chondrocytes treated as above, detected by WB. (C) The fluorescence of p65 in chondrocytes treated as above (scale bar: 20 μm). The data present as averages ± SD. ##*p *< 0.01, **p < 0.01, *n* = 6

To evaluate the mechanism of Ssn B, NF‐κB signalling, the classic inflammation‐associated pathway, was examined by western blot analysis. PA remarkably caused the upregulation of p‐IκBα and p‐p65 and promoted the degradation of IκBα. Nonetheless, these effects were remarkably suppressed by Ssn B pretreatment at the concentration of 10 μM (Figure 3A and B). We performed p65 immunofluorescence staining to assess NF‐κB signalling activity in PA‐treated chondrocytes. The chondrocytes were clustered into 3 groups: control, PA and PA + Ssn B. In control group, p65‐positive spots were mainly located in the cytoplasm. Nevertheless, after PA treatment, the p65 was remarkably translocated into the nucleus. Ssn B pretreatment mitigated p65 translocation (Figure 3C). Altogether, these data illustrated a suppressive effect of Ssn B on the activity of NF‐κB signalling in PA‐treated chondrocytes.

### 3.5 Influences of Ssn B on the interaction of TLR4/MD‐2 axis in PA‐stimulated human osteoarthritic chondrocytes

**FIGURE 4:**
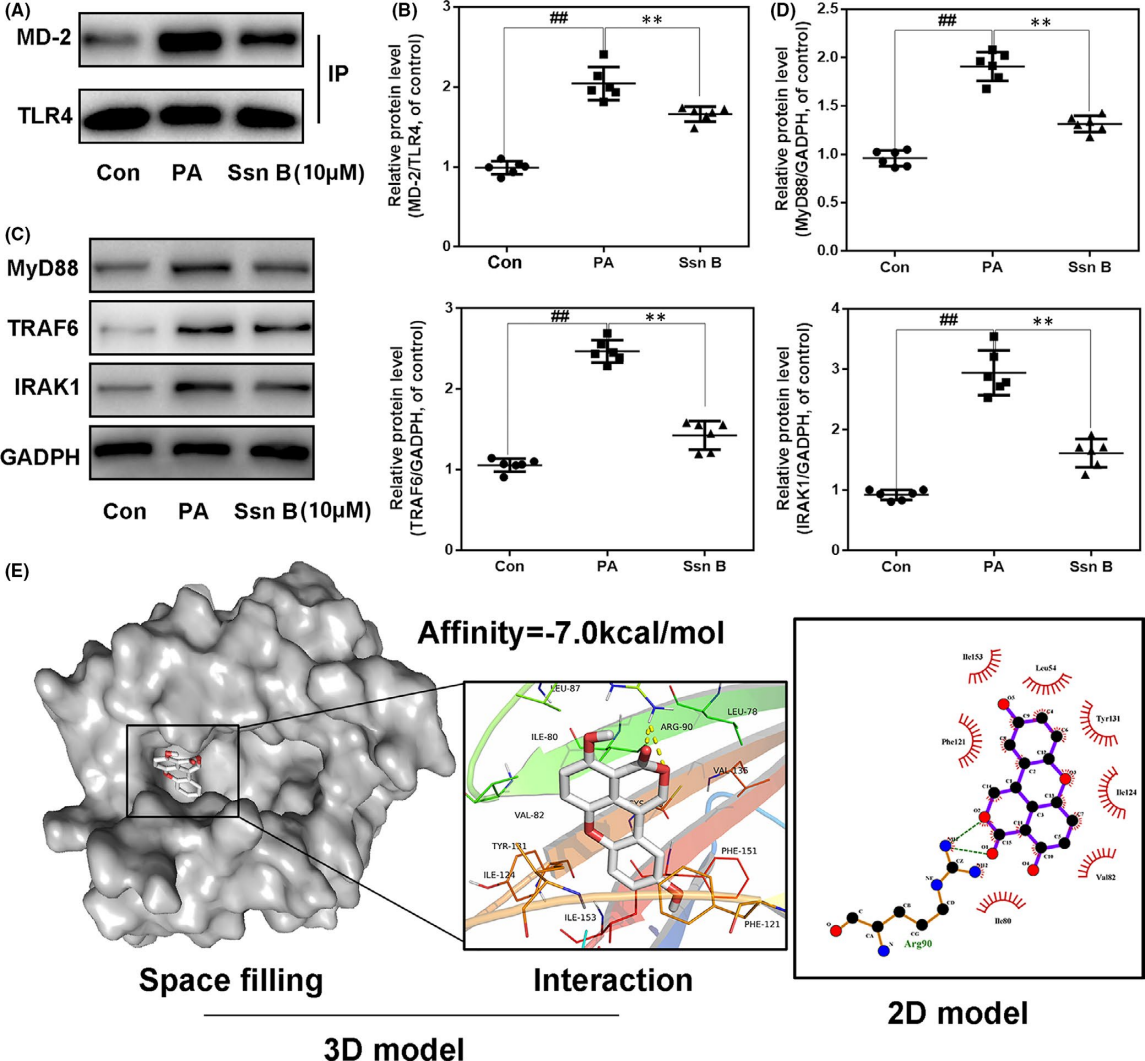
Effect of Ssn B on PA‐induced TLR4 activation. (A, B) The production of TLR4/MD‐2 complexes in chondrocytes treated as above, detected by immunoprecipitation. (C, D) The expression of MyD88, TRAF6 and IRAK1 proteins in chondrocytes treated as above, detected by WB. (E) The docking result of MD‐2 protein and Ssn B molecule. The data present as averages ± SD. ##*p *< 0.01, **p < 0.01, *n* = 6

TLR4 is a widely investigated upstream biomolecules of NF‐κB signalling. Nonetheless, the natural TLR4 agonists like PA do not directly dock to the receptor but are mediated by MD‐2. The crystal structures of TLR4 ectodomain, MD‐2 and PA show that 5 hydrophobic carbon chains of the lipid A bind within the hydrophobic pocket of MD‐2. To assess the effect of Ssn B on TLR4/MD‐2/NF‐κB axis, we firstly performed the co‐immunoprecipitation to investigate Ssn B’s exposure to the generation of the TLR4/MD‐2 complex under the PA stimulation. As shown in Figure 4A and B, PA facilitate the crosstalk of TLR4 with MD‐2, while Ssn B exposure suppressed this complex formation, which indicated that the Ssn B occupied the docking position of PA on the TLR4/MD‐2 complex. Furthermore, PA‐induced TLR4 activation is reported to be MyD88‐dependent and followed by the increase in multiple toll adapter proteins consisting of MyD88, TRAF6 and IRAK1, which are core factors to trigger the NF‐κB‐associated inflammation. Here, to determinate the effect of Ssn B on these signalling cascades, we performed western blot to evaluate the expressions of MyD88, TRAF6 and IRAK1 in PA‐induced human OA chondrocytes. PA treatment enhanced the level of MyD88, IRAK1 and TRAF6. Nevertheless, Ssn B remarkably repressed the expression of these toll adapter proteins (Figure 4C and D). Finally, we performed docking analysis on the binding of Ssn B and MD‐2 protein structure, the results revealed a high affinity of −7.0 kcal/mol of Ssn B with MD‐2 structure and the space filling model exhibited that the Ssn B was entirely embedded in the inhibitory pocket of MD‐2. In addition, local interaction of protein residue with Ssn B revealed a pivotal hydrogen bond was formed between Ssn B and Arg90 residue of MD‐2. What's more, the 2D view was conducted to show several hydrophobic bonds exist between Ssn B and Leu54, Ile153, Tyr131, Phe121, Ile124, Val82 as well as Ile80 (Figure 4E). Taken together, these data suggested that anti‐inflammation of Ssn B in chondrocytes might through TLR4/MD‐2/MyD88‐dependent pathway.

### 3.6 Influences of Ssn B on obesity ‐related OA in mouse

**FIGURE 5:**
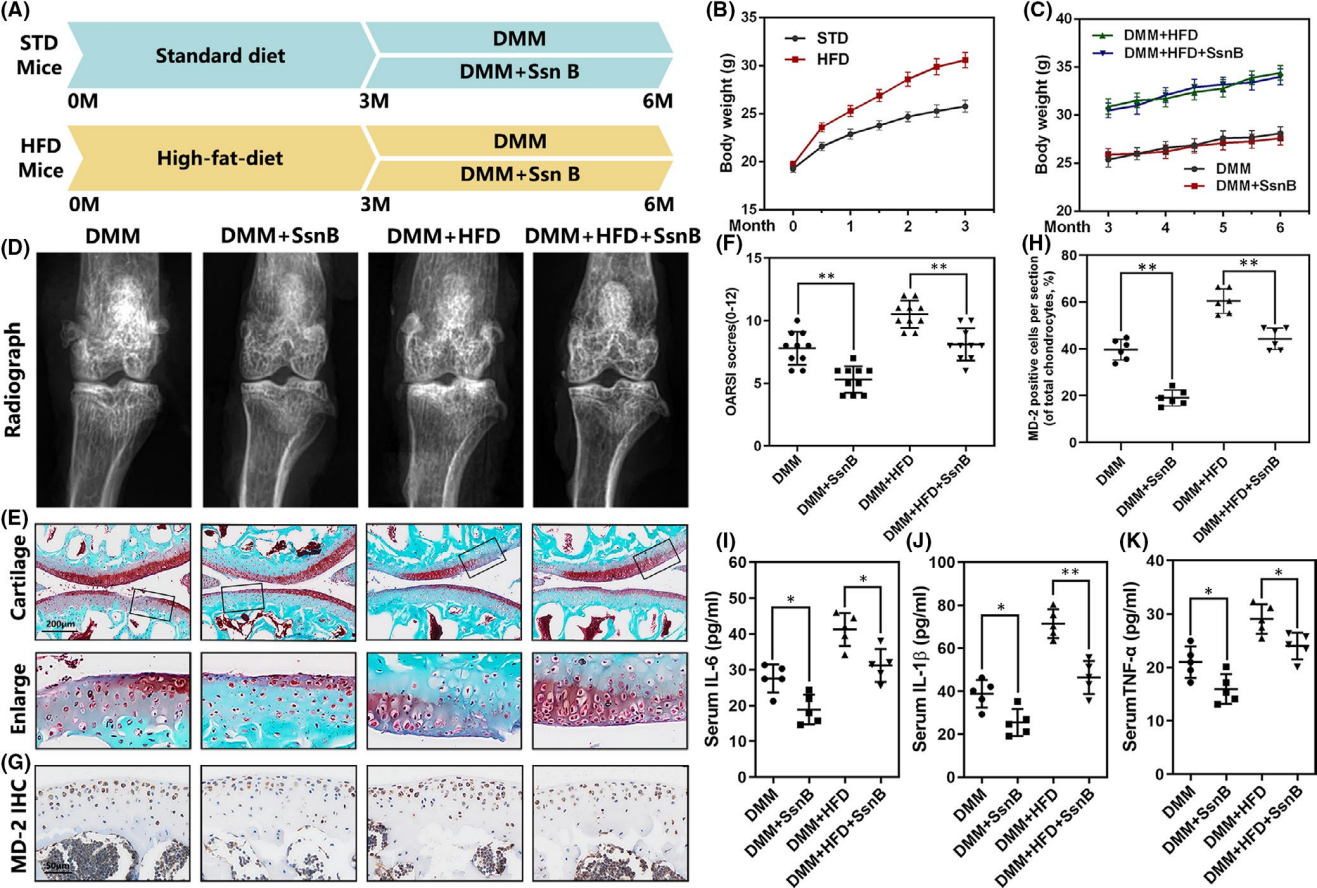
Ssn B prevents the development of obesity‐related OA in vivo. (A) The timeline graph indicates that the mice received treatments. (B) The body weight of mice in each group from 0 to 3 months. (C) Body mass of mice treated as above from 3 to 6 months. (D) X‐ray representative images of the knee joints of mice in each group. (E) Representative SO staining of cartilage from different experimental groups at 3 months post‐surgery. (F) Diagrams showed the OARIS scores of cartilage. (G, H) Representative MD‐2 IHC staining of cartilage from different experimental groups at 3 months post‐surgery. (I–K) Serum IL‐1β, IL‐6 and TNF‐α levels of mice in each group were detected by ELISA. The data present as averages ± SD. *p < 0.05, **p < 0.01

We developed the mouse obesity‐related OA model by feeding mice with HFD and surgically removing medial meniscus ligament to explore the role of Ssn *in vivo*. The timeline for animal processing is indicated in Figure 5A. By weighing regularly, we found that HFD‐fed mice were significantly heavier than the STD‐fed mice, within 0.5 to 3 months (Figure 5B). From the 3rd to 6th month, the weight of the mice fed by HFD continued to increase, while the mice in the STD group maintained a weight of about 25g (Figure 5C). Notably, regardless of the STD‐fed or HFD‐fed mice, Ssn B treatment has not changed the weight of mice. All animals were sacrificed 6 months later to collect knee joint tissue for further evaluation. As for the radiograph, DMM group showed excessive narrowing of the joint space and osteosclerosis occurs in the load‐bearing area of the tibial plateau, which was more obvious in the HFD + DMM group (Figure 5D). But these phenomena were alleviated after Ssn B administration. In SO staining, we found that HFD feeding further aggravated the destruction of cartilage structure with rougher surface, fewer red areas and a higher OARSI score, suggesting that HFD could accelerate DMM‐induced cartilage degeneration in mice. Nevertheless, these pathological changes were ameliorated to varying degrees in Ssn B‐treated mice (Figure 5E and F). Based on immunohistochemical staining, there are more MD‐2‐positive chondrocytes in cartilage tissue of HFD‐fed mice, but Ssn B treatment could alleviate this phenomenon (Figure 5G and H). Detecting serum IL‐6, TNF‐α, as well as IL‐1β levels by ELISA, we found that Ssn B administration reduced the serum inflammatory factor levels in OA mice and obese OA mice (Figure 5I–K).

## 4 DISCUSSION

Obesity is causally linked to joint disease.^6^, ^35^ The abnormal lipid accumulation not only increased body mass, leading to a heavy mechanical stress to the joint, but also is responsible for the pathological development of OA.^36^ Firstly, for obese patients, the risk of developing arthritis in nonweight‐loading joints was also increased.^7^, ^37^, ^38^ Secondly, the high‐fat diet‐mediated obese mice present a significant joint degeneration and systemic inflammation, while it is reported that escalated mechanical loading of joint via exercise did not enhance OA, but rather ameliorate OA.^39^ Therefore, the lipotoxicity resulting from dysregulated lipid metabolism in OA development should be seriously considered. Notably, lipids (including FFAs) are demonstrated to be accumulated in the cartilage and the synovial fluid.^11^, ^12^, ^13^ The free fatty acids could induce the secretion of pro‐inflammatory and pro‐apoptotic mediator in chondrocytes and synoviocytes of OA patients in *in vitro* and *in vivo* study.^10^ Meanwhile, PA co‐culture caused expressions of COX­2 and IL‐6 in human chondrocytes and promoted ECM degradation in cartilage explants.^40^ Therefore, in the present study, we applied PA as an *in vitro* stimulant to mimic the inflammatory environment caused by FFA in human osteoarthritic chondrocytes to explore a novel therapeutic method for the treatment of obesity‐related OA.

It has been documented that traditional Chinese medicine exerts chondroprotective effects by suppressing inflammatory response and possesses the potential to treat OA.^41^ However, no such agents have been reported to be applied in targeting obesity‐related OA. Herein, we demonstrated that Ssn B, a core bioactive component of the traditional Chinese herb *S*.*stoloniferum*, as a novel TLR’s inhibitor, significantly inhibits PA‐mediated inflammation in chondrocytes. In addition, we explored the specific protection mechanism of Ssn B.

Free fatty acids, particularly PA, were reported to be associated with chronic inflammation in chondrocytes.^10^, ^40^ Specifically, PA could promote pro‐inflammatory cytokine production via activating NF‐κB signal.^42^ With the stimulation of PA, the IκBα is phosphorylated and then frees and initiates the p65 translocated from the cytoplasm to the nucleus; IκBα is consequently degraded in the cytoplasm. p65 in the nucleus promotes the transcription of related gene of catabolic enzymes and inflammatory mediators.^19^ Nitric oxide and PGE2 are two major inflammatory productions, catalysed by iNOS and COX‐2, respectively, which not only improve the release of MMPs and ADAMTS, but also disturb collagen II and proteoglycan synthesis, leading to the ECM degradation and ultimate cartilage corrosion.^29^ All of these factors mentioned above, together with TNF‐α and IL‐6, accelerate the OA progression. Herein, we found that the overproduction of PGE2 and nitric oxide and the upregulation of COX‐2 and iNOS are remarkably repressed by Ssn B in PA‐administrated human OA chondrocytes. Consistent findings were also seen in the expression of TNF‐α and IL‐6. Besides, PA‐triggered activation of NF‐κB in chondrocytes was suppressed by Ssn B. These data are consistent with the works of wang et al. They explored the anti‐inflammation of Ssn B in adipocytes by targeting NF‐κB inhibition.^25^ Moreover, Ssn B was also shown an inhibitory effect on the generation of MMP‐13 along with ADAMTS‐5 and diminished the degradation of type II collagen and aggrecan in human OA chondrocytes under the PA stimulation. These data indicated that Ssn B has potential for the therapy of OA.

Despite numerous cascades are regarded as upstream targets for NF‐κB‐involved inflammation, the TLR4 signalling cascade is one of the most widely studied pathways being closely linked to FFA‐correlated inflammation.^16^ The classic TLR4 pathway involves MyD88‐dependent and MyD88‐independent cascades that stimulate signalling through TRIF (TIR domain‐containing adaptor‐inducing interferon‐β). Of note, PA‐induced inflammation was reported to be MyD88‐dependent.^17^ In brief, upon PA‐triggered TLR4 dimerization, the bridging adaptor MyD88 adaptor­like (MAL; also referred to as TIR domain‐containing adaptor protein, or TIRAP) mobilizes MyD88 resulting in its polymerization and interaction with several IRAKs and then autophosphorylate and activate TRAF6, which followed by the stimulation of the central pro‐inflammatory transcription factor NF­κB to trigger the subsequent inflammatory responses.^16^ Actually, MD‐2 is the key participant to construct the TLR4/MD‐2 complex to mediate the deleterious response during several DAMPs like LPS and PA‐induced TLR4 activation.^18^, ^43^ In the current study, we firstly conduct Co‐IP to find that Ssn B decreased the interaction between TLR4 and MD‐2 and downregulated the expression level of the toll adapter proteins (Myd88, TRAF6 and IRAK1) under PA stimulation, which was consistent with the results of MD‐2 docking analysis. The binding model for MD‐2 showed that Ssn B can directly occupied its inhibitory binding pocket. TLR4/MD‐2/Myd88 pathway participates in the protective effect of Ssn B on PA‐induced OA chondrocytes.

**FIGURE 6:**
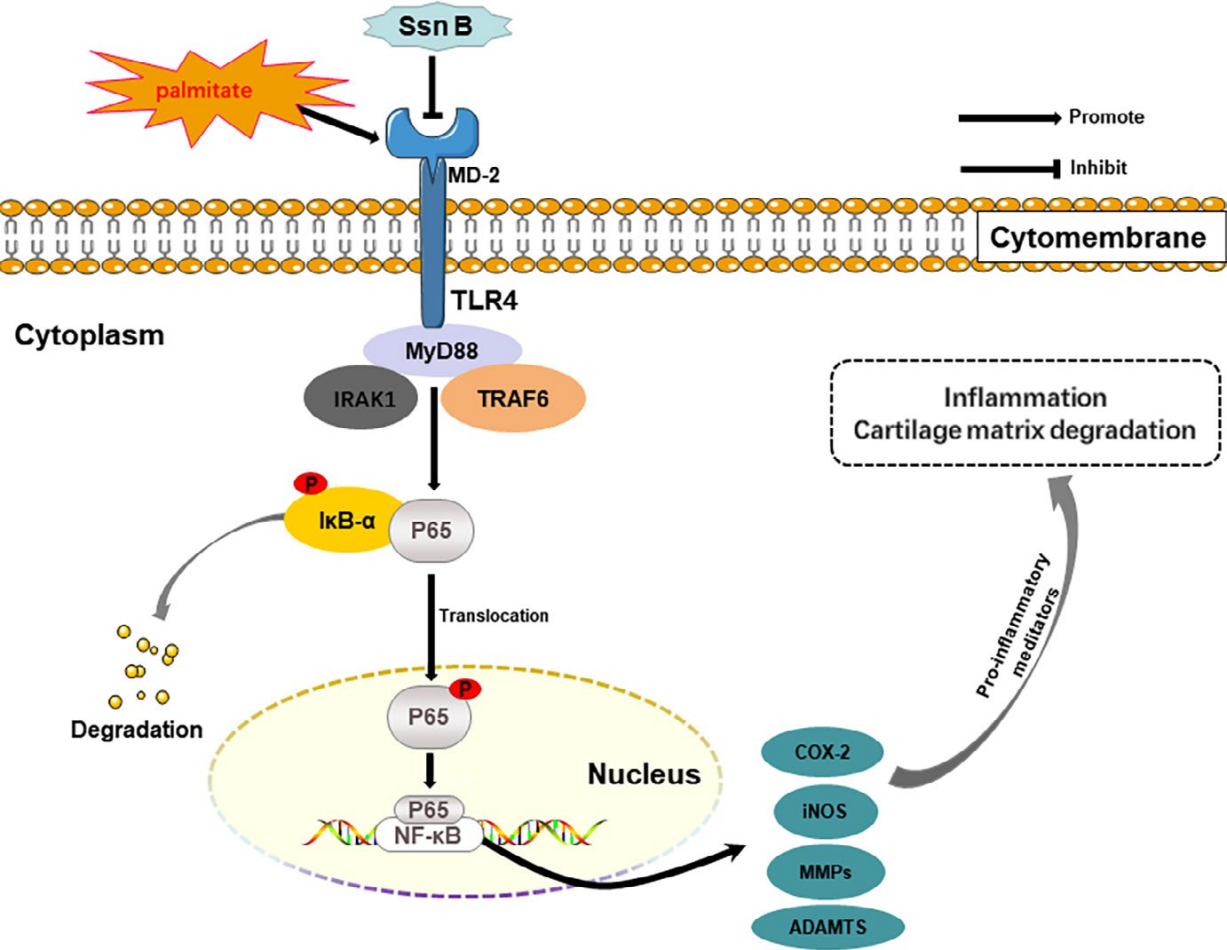
Schematic illustration of the potential protective effects of Ssn B in osteoarthritis development

In summary, we demonstrated that Ssn B exerts protective effects against inflammation and matrix degradation in PA‐induced OA chondrocytes by inhibiting TLR4/MD‐2/MyD88‐mediated NF‐κB signalling cascades (Figure 6). Meanwhile, Ssn B alleviated the development of obesity‐linked OA in mice. Thus, Ssn B represents a prospective agent for treating the obesity‐related OA.

## CONFLICT OF INTEREST

The authors declare that they have no conflict of interest.

## AUTHOR CONTRIBUTION

**Enxing Xue:** Conceptualization (equal); Writing – review & editing (lead). **Haiwei Ma:** Data curation (equal); Methodology (equal); Visualization (equal); Writing – original draft (lead). **Chenglong Xie:** Data curation (equal); Methodology (equal); Visualization (equal). **Gaolu He:** Data curation (equal); Writing – review & editing (supporting). **Zhengtai Chen:** Data curation (equal); Software (equal); Visualization (equal). **Hongwei Lu:** Data curation (equal); Visualization (equal). **Hongqiang Wu:** Software (equal); Validation (equal). **Hanchen Cai:** Methodology (equal); Software (equal). **Zihan Dai:** Visualization (equal). **Baolong Lii:** Formal analysis (equal). **Cong Xu:** Conceptualization (supporting); Project administration (lead); Validation (equal).

## Data Availability

Data available on request from the authors.
